# How should electronic health records be designed? A cross-sectional study in patients with psoriasis

**DOI:** 10.1186/s12911-019-0926-5

**Published:** 2019-11-12

**Authors:** Toni Maria Klein, Matthias Augustin, Marina Otten

**Affiliations:** Institute for Health Services Research in Dermatology and Nursing (IVDP), University Medical Center Hamburg-Eppendorf (UKE), Martinistraße 52, 20246 Hamburg, Germany

**Keywords:** Electronic health record, EHR, Acceptability, Psoriasis, Dermatology, Skin, Patients, Patients’ perspective

## Abstract

**Background:**

Electronic health records (EHRs) are promising tools for routine care. These applications might not only enhance the interaction between patient and physician but also support therapy management. This is crucial in complex and chronic conditions like psoriasis. However, EHRs can only unfold their full potential when being accepted by the users. Therefore, this study aims to analyse how EHRs should be designed for patients with psoriasis and to identify differences between patient subgroups.

**Methods:**

We developed a questionnaire on the acceptability of EHRs based on literature research and results from focus groups. Participants completed a paper-based or electronic version of the questionnaire. We recruited participants at an outpatient clinic as well as online via patient associations and a social media platform. We analysed data using descriptive statistics and bivariate analyses applying Chi-square and Fisher’s exact test.

**Results:**

The sample encompassed 187 patients with psoriasis. Data reveals that 84.4% of the participants can think of entering data into an EHR. Participants prefer entering data at home (72.2%) instead of entering data in the waiting room (44.9%) and using an own internet-ready device (laptop/computer: 62.6%; smartphone/tablet: 61.5%) instead of a provided device (46.0%). Altogether, 55.6% of participants would accept entering data on a monthly basis when this lasts between one and 10 minutes and further 27.8% would accept even longer lasting data entry. Data privacy is of great concern (e.g. patient should decide who has access to data: 96.7%). Subgroup analyses reveal differences with regard to age, educational level, burden due to psoriasis, number of internet activities, use of electronic questionnaires and mode of administration.

**Conclusion:**

The high acceptance of entering data is favourable for the implementation of EHRs. The results suggest technical and structural recommendations: Differences between subgroups support the development of flexible EHRs encompassing a basic module, which is expandable with further add-ons, and compatible to different devices. Furthermore, involving patients by entering data into an EHR requires that physicians communicate open-mindedly with the patient and consider data throughout decision-making. Patients should remain owner of their own health data and decide about its processing.

## Background

Electronic health records (EHRs) are developed with different features and implemented in various settings [1], but are used uniformly to enter, store, and monitor patient data. In Germany it is politically intended to implement EHRs comprehensively [2]. The design of EHRs can range from an electronic folder collecting scanned paper documents to a system allowing for digital data entry and storage including automated analyses and alert functions [3]. Depending on the specific design patients’ degree of involvement differs in the process of data management. While some systems are only accessible by clinicians and healthcare staff [4], in others the patient reads the physician’s notes [5, 6] or enters information into the EHR [7, 8]. Latter forms put the patient into the place of an active stakeholder in the process of care delivery. Such forms of patient involvement in the treatment process are in line with the principle “nothing about me without me” [9] and a major demand in patient-centered healthcare systems [10]. Hence, focusing on the patient is crucial when developing and implementing new technological applications. Finally, patients will only accept and use EHRs when they meet their expectations, needs, and routine [11].

Benefits of EHRs can only emerge when actually being utilized [12]. Patients’ confidence in utilization of an EHR increases over times when frequently using this application [6]. Utilization could be facilitated by easy accessibility, compatibility for different devices, and availability of support systems [13]. Nevertheless, patients’ preferences regarding the design and technical realization of an EHR differ with regard to patient characteristics [8].

Nohl-Deryk et al. [14] identify patients as driving force for the use of eHealth applications, which can be supported by studies revealing patients’ overall high acceptance towards and positive experiences with the use of EHRs [6, 7, 13, 15]. Patients report beneficial effects, such as that EHRs improve the understanding of both health problems and treatment decision, serve as memory aids, empower the patient, and make their consultation more efficient [6, 7, 13]. Additionally, improvements in the interaction between patients and physicians can be detected such as enhanced patient-physician-communication and enriched collaborative treatment processes [7, 16, 17]. Furthermore, patients experience that physicians better understand individual concerns due to reading patients’ data entries as this facilitates preparing and setting priorities according to the needs of each patient [7, 15]. However, patients do not expect overall time savings for physicians due to the use of EHRs [15]. Nevertheless, reduction in administrative burden and improvements in data quality could be identified [18]. Overall, patients’ broad acceptance makes EHRs promising tools.

Electronic processing of health data makes data privacy a great issue. Accordingly, more than one third of patients express concerns regarding data privacy when implementing an EHR [5]. Additionally, access rights need to be assigned carefully, especially when EHRs are designed to enhance the inter-institutional transition of medical data. Mairoana et al. [19] found that patients are mostly willing to assign access rights to primary care providers and other clinicians. This corresponds with findings by Hanna et al. [13] revealing that health data in EHRs might be most useful when new physicians have access to these information. However, on-going experience reduces concerns about data privacy by more than half [5].

In particular, the treatment of chronic conditions can largely profit from using EHRs by, among others, increasing patient self-management and improving patient-provider interaction [20]. This also accounts for psoriasis, which is a disease causing painful, itching and stinging skin lesions [21] and leading to enormous costs for the patient, the healthcare system, and the economy [22]. The aetiology of psoriasis is still uncertain, despite there is evidence for genetic dispositions [23] as well as external triggers and autoimmune reactions provoking the disease [24]. Multiple comorbidities are associated with psoriasis, such as rheumatoid arthritis, Crohn’s disease, diabetes mellitus, ischemic heart disease, hyperlipidaemia, and hypertension [25]. Also psychological conditions, like depressions, anxiety, and suicidality, are more prevalent in patients with psoriasis [21]. Psoriasis affects all areas of life [26] and reduces patients’ quality of life [27]. The impact on patients’ well-being, the high rates of comorbidities, and the variety of possible trigger factors make psoriasis a complex and difficult to manage condition. The World Health Organization outlines the necessity to adequately monitor the progress of psoriasis and its treatment including the use of standardized tools and guidelines as well as inter-professional collaboration [21]. Therefore, patients with psoriasis might greatly benefit from EHRs when integrating both information on patients’ general health and tools specifically for the treatment of this disease. Due to the complexity and chronicity of the diseases, patients with psoriasis are fully involved in the treatment and would also need to participate in the maintenance of the EHR. However, we could not identify a single study on the acceptability of EHRs focussing on dermatological patients with psoriasis.

The purpose of this study is to analyse how EHRs should be designed for patients with psoriasis. Specifically, we investigate distinct functionalities of EHRs, such as data entry, access rights to health data, and data privacy. Furthermore, we identify differences between patient subgroups. The pre-interventional design of this study allows to develop EHRs that are usable and acceptable by patients.

## Methods

This is a prospective, cross-sectional study assessing the patients’ perspective, which we conducted in compliance with the STROBE statement.

### Study population and procedure

This study surveyed patients with psoriasis, who were German speaking and gave written informed consent. Patient recruitment took place via different pathways to include a diverse patient sample: Patients at the outpatient care unit of the University Medical Center Hamburg-Eppendorf (UKE) completed a paper-based questionnaire during their waiting time. Furthermore, participants accessed the electronic questionnaire via a group for patients with psoriasis on the social media platform Facebook encompassing almost 9000 members and via the websites of two German psoriasis associations (“Psoriasis-Netz”, “Deutscher Psoriasis Bund e. V.”). The latter association additionally distributed the electronic questionnaire via a mailing list of 300 patients. Targeted sample size was at least 50 participants as this allows for conducting regression analyses [28]. However, for approaching the research question in the present manuscript we only applied descriptive and bivariate statistics. Data collection lasted from July to August 2018.

### Questionnaire

We developed the questionnaire based on both literature research and the analysis of previously conducted focus groups of patients with psoriasis. Literature research revealed that previous studies mostly assessed acceptance of functionalities of EHRs during- and post-interventional. In three focus groups 14 patients discussed patient-reported outcomes, decision-making processes, and the use and maintenance of EHRs, among others. All focus groups were transcribed and subjected to content analysis (more details about the procedure and analysis of the focus groups will be published elsewhere). Results from focus groups raised further possible functionalities of EHRs and augmented findings from literature research. Studied participants might have experience with using electronic questionnaires but not with using EHRs, as no EHR has been implemented in Germany, yet. Therefore, the questionnaire asked for the patients’ acceptance of possible features identified during literature research and focus groups, depicting an EHR allowing for data analysis and visualization as well as being maintained by both patient and physicians.

The developed questionnaire underwent cognitive debriefing to test clarity of wording. The questionnaire (see Additional File 1) was developed in German and finally encompassed the following topics: (a) sociodemographic data, (b) disease-specific data, (c) media use, (d) attitude towards patient-reported outcomes, (e) attitude towards entry of data into EHRs, (f) attitude towards EHRs, and (g) attitude towards visualization of outcomes. Within topics (a) to (c) response options of the items presented single-choice, multiple-choice and free text answers. Within topics (d) to (g) response options presented various 5-point Likert-type scales, added by a sixth response category ‘*does not matter’* for items on data privacy within topic (e). For this article, we analysed data on the topics (a), (b), (c), and (e). Questionnaires of patients not completing any item of topic (e) were excluded from the analysis.

### Statistical analysis

We used IBM SPSS Statistics Version 23 for statistical analysis. We present descriptive statistics for continuous scaled (mean, standard deviation, median), ordinal, and nominal items (frequency distribution, percentages). For items with less than 5% missing values, we exclude cases with missing values from analysis, whereas for variables with more missing values we present figures based on the total sample. For multiple-choice items about comorbidities and internet activities, we counted the number for each patient. We categorized patients as having high or low burden due to psoriasis. As no clinical data on the disease was available, indicators for being highly burdened were reporting at least monthly consultations at a dermatologic specialist, a dermatological ambulatory clinic, or an in-patient setting or reporting at least three comorbidities. In order to conduct subgroup-analyses, we condensed 5-point Likert-type scaled items into three categories, representing one positive (e.g. (totally) agree), one neutral or undecided (e.g. neither nor), and one negative (e.g. (totally) disagree) category.

We conducted subgroup analyses with regard to patients’ gender (male/female), age (≤ 40 years/41 to 60 years/≥ 61 years), educational background (low/middle/high), burden of patients (low/high), number of internet activities (0–3 activities/4–6 activities/7–9 activities), experience with using electronic questionnaires (yes/no) and mode of administration (paper-based/electronic) using cross tables. For analyzing differences, we conducted Chi-square (Χ^2^) and Fisher’s exact tests (FET). In the text, we present FET when more than 20% of the cells in the respective cross table had expected counts below five [29]. We applied a significance level of α = 0.05. Additionally, we calculated adjusted standardized residuals to investigate the direction of association, revealing significance with values below − 1.96 and above 1.96.

## Results

### Descriptive analyses

A total of 190 patients participated in the study, of which three were excluded due to previously defined exclusion criteria (not completing items of topic (e)). Hence, the final sample encompasses 187 participants, with 27.3% (*n* = 51) completing the paper-based and 72.7% (*n* = 136) completing the electronic questionnaire. The sample consists of slightly more men (*n* = 93, 51.4%). Mean age is 51.62 years (SD = 15.26, Mdn = 55.00, Min = 15.00, Max = 93.00). Table 1 and Table 2 display the sample’s characteristics. The most frequently reported comorbidities are psoriasis arthritis (*n* = 73, 39.0%), cardiovascular diseases (*n* = 53, 28.3%), and obesity (*n* = 29, 15.5%). The participants’ most frequent internet activities are searching for information (*n* = 174, 93.0%), writing and reading e-mails (*n* = 171, 91.4%), as well as online shopping and online banking (each *n* = 133, 71.1%). Three participants (1.6%) report no internet activities at all. Almost one quarter (*n* = 43, 23.0%) of patients reports already having used electronic questionnaire when visiting their physicians.

**Table 1:** Frequencies and percentages of sample characteristics

	Total [n] (missing [n])	Frequency [n]	Percentage [%]
Questionnaire Mode	187 (0)		
Paper-based questionnaire		51	27.3
Electronic questionnaire		136	72.7
Gender	181 (6)		
Male		93	51.4
Female		88	48.6
Education	183 (4)		
Low		30	16.4
Middle		53	29.0
High		100	54.6
Frequency of consultations	185 (2)		
At least monthly		24	13.0
Less frequently than monthly, at least quarterly		98	53.0
Less frequently than quarterly		63	34.0
Current treatment setting (multiple choice)	187 (0)		
General practitioner		35	18.7
Dermatologic specialist		89	47.6
Dermatologic ambulatory clinic		73	39.0
In-patient setting		2	1.1
No treatment		24	12.8
Burden due to psoriasis	187 (0)		
Low		136	72.7
High		51	27.3
Daily use of smartphone*	171 (16)	157^†^	84.0
Daily use of computer*	150 (37)	71^†^	38.0
Daily use of tablet*	137 (50)	55^†^	29.4
Daily use of laptop*	146 (41)	50^†^	26.7
Daily use of any device	179 (8)	173^†^	96.6
Experience with paper-based questionnaire	185 (2)	153^†^	82.7
Experience with electronic questionnaire*	175 (12)	43^†^	23.0

Cases with missing values are excluded from analysis. *variable with > 5% missing values: total percentages (basic values include missing values) are displayed; ^†^amount of participants reporting use

**Table 2:** Mean, standard deviation, median, and range of sample characteristics

	total [n] (missing [n])	Mean	SD	Mdn	Range
Age [years]	184 (3)	51.62	15.26	55.00	[15.00; 93.00]
Time since initial symptoms [years]	186 (1)	28.03	17.49	26.50	[0.00; 73.00]
Time since initial diagnosis [years]	184 (3)	26.03	17.10	25.00	[0.00; 72.00]
Comorbidities [n]	187 (0)	1.24	1.29	1.00	[0.00; 5.00]
Internet-activities [n]	187 (0)	5.45	1.99	6.00	[0.00; 9.00]
Health-related internet activities [n]	187 (0)	2.93	1.55	3.00	[0.00; 6.00]

SD: standard deviation; Mdn: median

Participants can largely think of entering information into an EHR (*n* = 156, 84,8%). Most participants would agree on entering data at home (*n* = 135, 72.2%) and on using their own laptop/computer (*n* = 117, 62.6%) or smartphone/tablet (*n* = 115, 61.5%). Less participants would agree on entering data in the waiting room (*n* = 84, 44.9%) or on using a provided device (*n* = 86, 46.0%). Accepted duration of data entry was assessed for monthly, weekly, and daily entry. Monthly entry would be accepted by 55.6% (*n* = 104) if lasting between one and 10 minutes Additionally, more than one fourth (*n* = 52, 27.8%) would accept even longer duration of entry. Only 4.3% (*n* = 8) regard entering data on a monthly basis as too often, while 12.3% (*n* = 23) did not answer this item. Tables 3 and 4 display the detailed response behaviour.

**Table 3:** Frequencies and percentages of data entry and data privacy

	Total [n] (missing [n])	+ [n(%)]	O [n(%)]	- [n(%)]
Can you think of entering data into an EHR	184 (3)	156 (84.8)	10 (5.4)	18 (9.8)
Can you think of entering data at home*	166 (21)	135 (72.2)	6 (3.2)	25 (13.4)
Can you think of entering data in the waiting room*	158 (29)	84 (44.9)	10 (5.3)	64 (34.2)
Can you think of entering data using own smartphone/tablet*	164 (23)	115 (61.5)	8 (4.3)	41 (21.9)
Can you think of entering data using own laptop/computer*	158 (29)	117 (62.6)	8 (4.3)	33 (17.6)
Can you think of entering data using provided device*	158 (29)	86 (46.0)	10 (5.3)	62 (33.2)
Me as a patient should be able to decide who has access to my data	184 (3)	178 (96.7)	6 (3.3)	0 (0.0)
Me as a patient should know where and how my data is stored	181 (6)	181 (100)	0 (0.0)	0 (0.0)
Only eligible persons should be able to enter information about me	183 (4)	176 (96.2)	4 (2.2)	3 (1.6)
I would agree on providing my data for scientific/research purposes	183 (4)	171 (93.4)	6 (3.3)	6 (3.3)
Information needs to be stored and displayed with sufficient clarity, so it can be considered during treatment	182 (5)	177 (97.3)	0 (0.0)	5 (2.7)
Physician needs to actually consider data for decision-making	179 (8)	171 (95.5)	4 (2.2)	4 (2.2)

Cases with missing values are excluded from analysis. *variable with > 5% missing values: total percentages (basic values include missing values) are displayed; +: (totally) agree/applies; O: neither nor; −: (totally) disagree/not applies

**Table 4:** Frequencies and percentages of maximum duration for entering data

	Total [n] (missing [n])	Interval too often	1 min	5 min	10 min	20 min	30 min	> 30 min
		[n(%)]	[n(%)]	[n(%)]	[n(%)]	[n(%)]	[n(%)]	[n(%)]
Daily *	167 (20)	103 (55.1)	22 (11.8)	27 (14.4)	12 (6.4)	1 (0.5)	1 (0.5)	1 (0.5)
Weekly*	166 (21)	42 (22.5)	12 (6.4)	59 (31.6)	35 (18.7)	10 (5.3)	6 (3.2)	2 (1.1)
Monthly*	164 (23)	8 (4.3)	7 (3.7)	34 (18.2)	63 (33.7)	29 (15.5)	17 (9.1)	6 (3.2)

Cases with missing values are excluded from analysis. *variable with > 5% missing values: total percentages (basic values include missing values) are displayed

In general, participants highly value data privacy (see Table 3), as they would like to know where and how data is stored (*n* = 181, 100%), to decide who has access to their own data (*n* = 178, 96.7%), and that only eligible persons are able to enter data (*n* = 176, 96.2%). Nevertheless, the majority would provide data for research purposes (*n* = 171, 93.4%).

For the vast majority, storing data in sufficient clarity (*n* = 177, 97.3%) and actually considering data for decision-making (n = 171, 95.5%) are important conditions for entering any kind of data into an EHR, as depicted in Table 3.

Regarding clinical data, participants see physicians most often as eligible to enter data (*n* = 173, 93.0%), followed by patients themselves (*n* = 99, 52.9%). Participants regard other clinical staff less frequently as eligible for clinical data entry (*n* = 51, 27.3%).

Participants state most often that the physician mainly treating their psoriasis should always have access to psoriasis-related data (*n* = 155, 83.8%), followed by other physicians treating the psoriasis (*n* = 126, 68.5%). For other service providers, patients would give only limited access (only after patient’s permission or if patient is present). This accounts for other service providers treating the psoriasis (*n* = 121, 65.4%), other physicians not treating the psoriasis (*n* = 105, 57.4%) and other service providers not treating the psoriasis (n = 121, 65.4%). A certain share of participants would never allow other service providers not treating the psoriasis (*n* = 23, 12.3%) or their own health insurance company (*n* = 22, 12.0%) to access the data. However, 39.7% (*n* = 73) would allow their health insurance company to always access the data. Participants’ response behaviour on access to psoriasis-related data is displayed in Table 5.

**Table 5:** Frequencies and percentages of access to psoriasis-related data for different stakeholders

	Total [n] (missing [n])	Always	After permission	When patient is present	Never	Don’t care
		[n(%)]	[n(%)]	[n(%)]	[n(%)]	[n(%)]
Physician mainly treating patient’s psoriasis	185 (2)	155 (83.8)	29 (15.7)	0 (0.0)	0 (0.0)	1 (0.5)
Other physician treating patient’s psoriasis	184 (3)	126 (68.5)	52 (28.3)	3 (1.6)	1 (0.5)	2 (1.1)
Other service provider treating patient’s psoriasis	185 (2)	55 (29.7)	104 (56.2)	17 (9.2)	6 (3.2)	3 (1.6)
Other physician not treating patient’s psoriasis	183 (4)	66 (36.1)	90 (49.2)	15 (8.2)	7 (3.8)	5 (2.7)
Other service provider not treating patient’s psoriasis	185 (2)	34 (18.4)	94 (50.8)	27 (14.6)	23 (12.4)	7 (3.8)
Health insurance company	184 (3)	73 (39.7)	77 (41.8)	8 (4.3)	22 (12.0)	4 (2.2)

Cases with missing values are excluded from analysis

### Subgroup analyses

We analysed participants’ responses with regard to specific characteristics. Table 6 gives an overview of items for which participants’ response behaviour differ with regard to investigated characteristics. Gender is not included in the table, as it does not reveal significant differences. Detailed distributions of significant cross tables can be seen in the Additional File 2 Table S1 to Table S5 including frequencies, percentages, and adjusted standardized residuals.

**Table 6:** Differences in participants’ answers (significant *p*-values) with regard to participant characteristics

Variable	Age	Education	Burden due to psoriasis	Internet activities	Ever used electronic questionnaires	Mode of administration
How can you think of entering information into an EHR?						
At home	–	0.001^†^	0.010^†^	0.008^†^	0.039^†^	0.013^†^
In the waiting room	< 0.001^†^	–	–	0.006^†^	–	0.049*
Using own smartphone/tablet	0.012^†^	0.008^†^	–	< 0.001^†^	–	–
Using own laptop/computer	–	0.011^†^	–	0.001^†^	–	–
Using provided device	0.006^†^	0.009^†^	–	–	–	–
How many minutes should data entry last at maximum in order for you to conduct it...						
daily?	0.001^†^	0.026^†^	–	0.006^†^	0.032^†^	–
weekly?	–	–	0.035*	0.032*	–	–
monthly?	–	0.006^†^	0.031*	–	–	–
Should following stakeholder have access to information about your psoriasis?						
Other service providers treating psoriasis	–	–	–	–	0.011^†^	–
Physicians not treating psoriasis	–	0.018^†^	–	–	0.020^†^	–
Other service providers not treating psoriasis	–	–	–	–	0.007^†^	–
My health insurance company	–	0.014*	–	–	–	–
Me as a patient should be able to decide who has access to my data	0.006^†^	–	–	0.002^†^	–	–

* p-value according to Χ^2^-test (when < 20% of the cells in the according cross table have expected values < 5); ^†^ *p*-value according to Fisher’s exact test (when ≥20% of the cells in the according cross table have expected values < 5); − not significant *p*-values

Regarding the patients’ age, there are significant differences revealing that older participants compared to younger participants are less willing to enter data in the waiting room (*p* < 0.001, FET), using their own smartphone/tablet (*p* = 0.012, FET), or using a provided device (*p* = 0.006, FET). In contrast, younger patients are more often willing to enter data on a daily basis when this lasts between one and ten minutes (*p* = 0.001, FET). Moreover, younger participants are more often undecided whether patients should be able to decide who has access to their data (*p* = 0.006, FET).

Compared to participants with low or middle educational level, highly educated participants state more often that they would enter data using their own laptop/computer (*p* = 0.011, FET) but would less often enter data using a provided device (*p* = 0.009, FET). Additionally, more participants with high educational level regard daily entry as too often (*p* = 0.026, FET). Concerning access to their data, highly educated participants would less often allow other physicians not treating the psoriasis (*p* = 0.018, FET), or their health insurance company (Χ^2^ = 12.572, df = 4, *n* = 176, *p* = 0.014) to always access data.

Patients with low burden due to psoriasis are less often willing to enter data at home than highly burdened participants (*p* = 0.010, FET). Additionally, more patients with low burden regard weekly data entry as too often (Χ^2^ = 8.626, df = 3, *n* = 187, *p* = 0.035).

Participants reporting the lowest number of internet activities most often deny any kind of data entry (at home (*p* = 0.008, FET); in the waiting room (*p* = 0.006, FET), using own smartphone/tablet (*p* < 0.001, FET), using own laptop/computer (*p* = 0.001, FET)). Moreover, these participants would less often enter data weekly when this lasts one to ten minutes (Χ^2^ = 13.766, df = 6, n = 187, *p* = 0.032). Only participants with the highest number of internet activities state that they are undecided about whether patients should be able to decide who has access to their data (*p* = 0.002, FET).

Participants ever having used electronic questionnaires state more often that they would always allow service providers treating the psoriasis (*p* = 0.011, FET) as well as physicians (*p* = 0.020, FET) and service providers (*p* = 0.007, FET) not treating the psoriasis to access their data.

Participants completing the paper-based questionnaire at the outpatient clinic agree more often willing to enter data in the waiting room than those completing the electronic questionnaire (Χ^2^ = 7.882, df = 3, *n* = 187, *p* = 0.049). Conversely, participants completing the electronic questionnaire favour data entry at home (*p* = 0.013, FET).

## Discussion

This study investigated how EHRs should be designed for the treatment of patients with psoriasis and identified preferences and requirements for a successful implementation. Results indicate overall high patients’ acceptance of using EHRs in the treatment of psoriasis as illustrated by patients’ high willingness to enter data themselves. Participants demand that physicians consider their data in the treatment and wish to decide about the processing of the data. Important differences in the entry of and access to psoriasis-related data could be identified between different subgroups.

We identified high acceptance of monthly data entry lasting between one and ten minutes. Alike, Anderson et al. [7] report that most people take less than 10 min to enter their agenda into an EHR. However, in the present study more than one fourth of the participants would even accept longer duration for data entry. A feasible solution would be to develop a basic EHR including a set of minimum data and providing further add-ons to give the opportunity to enlarge the amount of data being considered. This allows to develop individual solutions for each patient. In the present study, no single device to access EHR could be identified that would be preferred by all participants suggesting that EHRs should be compatible for different devices. This was also elucidated by Hanna et al. [13]. A compatible EHR also meets patients’ demands of being able to use their own device instead of a provided one.

In this study, participants wish that physicians consider the entered data in the treatment process. Meeting this demand has great potential to enhance patient involvement and to enrich discussions between patients and physicians [16]. Finally, recognizing the benefits of patient involvement could further enhance patients’ willingness to enter data and contribute to the treatment process.

In our study, older patients are more sceptical about entering data into an EHR, which corresponds with results of another study revealing that older patients show lower levels of acceptance and require more help when using an EHR [8]. Moreover, our study shows differences in preferred devices and frequency of data entry with regard to patients’ level of education. This emphasizes the necessity to find individual solutions for different patients.

Moreover, we identified that patients with higher burden due to psoriasis are generally more willing to use EHRs. It can be assumed that these patients have the most complex disease progressions, are most severely impaired, and hence require the most comprehensive forms of therapy management [27, 30]. The high acceptance within this patient group allows to suggest that especially these patients would enter data conscientiously, which makes EHRs a promising tool for enhanced therapy management.

Besides sociodemographic and disease-related characteristics of the participants, patients’ affinity to use electronic devices reveals significant differences. Our results show that participants who report a high number of internet activities state a more positive attitude towards the use of EHRs. Additionally, patients in our study being experienced with using electronic questionnaires would more often provide access to various physicians and service providers. This supports findings that familiarity with a certain technology leads to higher levels of acceptability [5, 6, 8]. Therefore, a flexible EHR might not only be promising to account for differences between but also for changes within individuals. Patients being unfamiliar with the use of electronic applications could start with a basic form of the EHR. After patients’ habituation with the application, this could be supplemented with further add-ons allowing for a more comprehensive data collection. Additionally, our results show that participants completing the electronic questionnaire preferred data entry at home, whereas patients completing the paper-based questionnaire preferred data entry in the waiting room. Preferences of each subgroup correspond with the actual setting of completing our questionnaire. These results might not only support hypothesis of more familiarity leading to higher acceptance of technology but might also indicate that participants felt comfortable completing the questionnaire in their current situation. This suggests that the study posed acceptable effort for participants.

We not only investigated requirements for data entry, but also patients’ preferences about access rights to their psoriasis-related data. Even though we identified differences between subgroups, participants are generally more willing to assign access rights to physicians and other stakeholder, who are most directly involved in the treatment process. This corresponds with findings of Maiorana et al. [19] and might result from increased trust towards frequently contacted healthcare providers [31]. Together with the German law on eHealth [32], these results underline that patients should have health data at their disposal. However, patients’ ownership should not only be realized in the structural assignment of access rights but should be considered also during consultation, as demanded by Milne et al. [16].

### Strengths and limitations

The major strength of this study is its focus on a diverse patient sample due to few exclusion criteria and multiple recruitment pathways. Besides organizational and provider-related barriers and facilitators, patient-specific requirements are crucial to facilitate the fidelity of EHR implementations involving patient action. Even though this questionnaire has not been psychometrically tested, it provides several quality criteria: Development of the questionnaire was based on the results of focus groups, which facilitated to integrate various aspects of acceptability of EHRs and to approach the linguistic expressions of patients. Additionally, there were only few drop-outs due to discontinuation of completing the questionnaire. Furthermore, participants’ responses revealed no contradictory findings within the sample as well as consistency with results from previous studies, which confirms validity of the items. To the best of our knowledge, this is the first study investigating barriers and facilitators for the implementation of EHRs specifically for the treatment of patients with psoriasis.

Nevertheless, we need to outline some limitations. The generalizability of the results is limited due to the sampling strategy of this study. On the one hand, patients recruited in the outpatient clinic have probably more severe forms of psoriasis than patients with psoriasis do have on average. On the other hand, a large share of participants completed the online questionnaire suggesting that these patients might be more enthusiastic about new technologies. This assumption is supported by the significantly higher total number of internet activities in this subgroup. In the development of the questionnaire we aimed to keep content and wording as close as possible to the results of the focus groups. However, this might have led to some items whose wording was formulated one-sided leading to ceiling effects and reduced variance in participants’ responses (e.g. “Only eligible persons should be able to enter information about me”, “Physician needs to actually consider data for decision-making”). Additionally, we need to emphasize that we investigated attitudes and that attitudes do not necessarily result in the intended actions [33]. Accordingly, it can be expected that the rates of actual utilization of EHRs will not equal the rates of acceptability. Moreover, we used Likert-type items to assess the acceptability of different settings, devices, and durations of data entry. Even though this allows evaluating patients’ overall attitude concerning each of these options, it does not state a clear decision for one preferred option.

## Conclusion

Our approach of investigating facilitators and barriers pre-interventional lays a sound basis for future planning of implementing new interventions. This is especially promising for countries being in the planning phase for the implementation of EHRs. However, it can also enrich the process of other countries already being in the implementation phase or in the revision of EHRs. Overall, the study reveals patients’ high willingness to enter health data into an EHR. Hereby, our study in a sample of patients with psoriasis reveals similar results as other studies in other patient samples with different conditions. In addition, we identified that high psoriasis-specific disease burden increase patients’ acceptability.

**Fig. 1:**
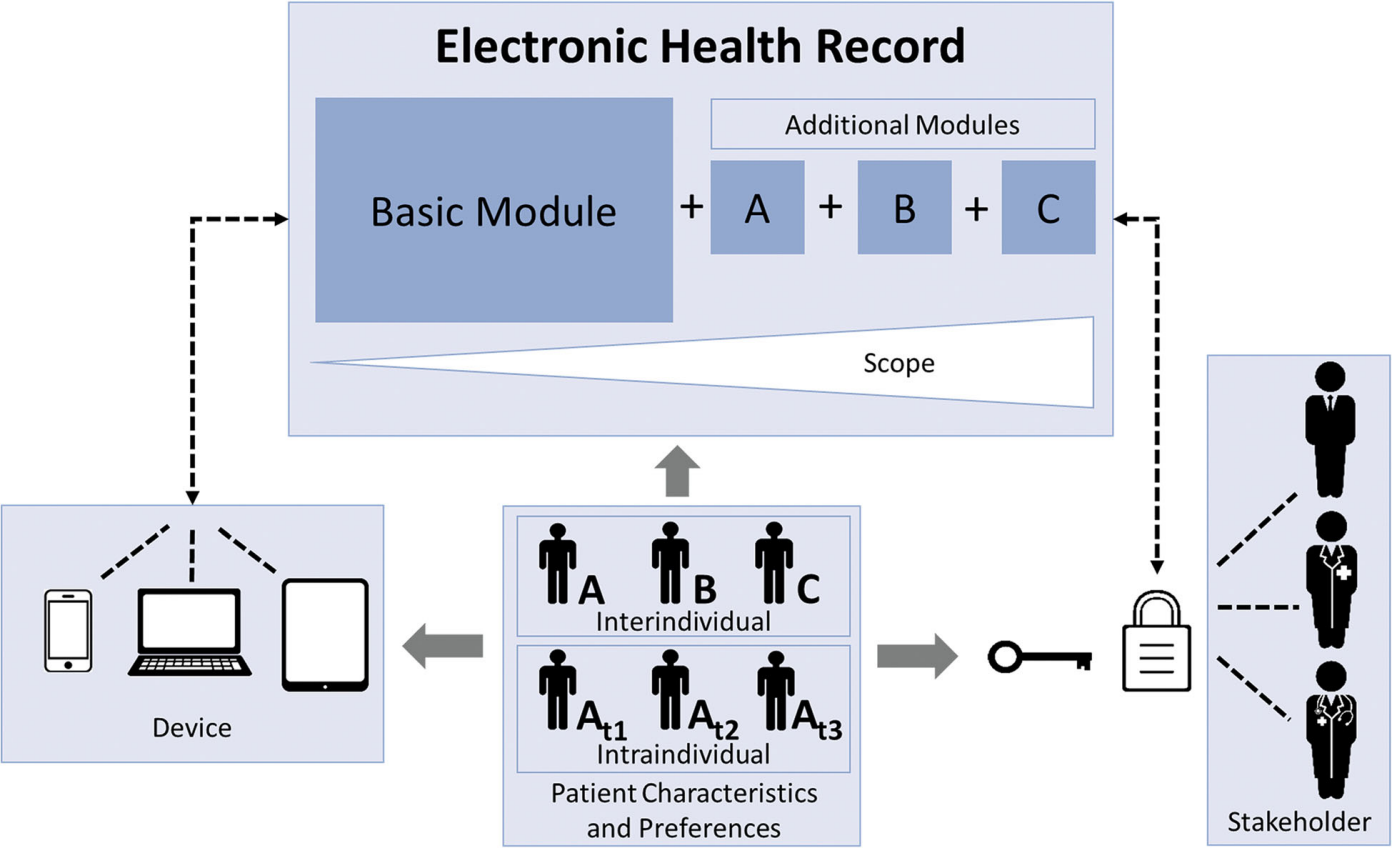
Model of recommendations for the development of electronic health records

From our results, we draw several technological and structural recommendations that are displayed in the model in Fig. 1. Primarily, we suggest developing a flexible EHR with a basic module, which is expandable according to the demand. This facilitates to develop individual solutions for each patient, as well as to adapt the EHR during the course of a treatment process. Additionally, developing systems that are compatible for different devices facilitate convenient utilization of the application and, hence, could increase the usage rates. Moreover, the individual patient should remain the owner of his or her health data and decide which stakeholder has access to this data. Additionally, data privacy and data security need to be guaranteed. Finally, physicians need to actively use data from the EHR within treatment and within common decision making.

In this study, we investigated patients’ attitudes. This perspective is crucial in electronic processing of health data. Nevertheless, the implementation of EHRs requires a comprehensive approach including all stakeholders. Physicians’ perspective may not be neglected, as they need to accept changes that will appear in their workflow as well as in their communication with the patient. Hence, physicians recognizing the beneficial consequences of EHRs can largely enhance the implementation process.

## Supplementary information

**Additional File 1.** Patient questionnaire (German original language).

**Additional File 2.** Tables S1-S5 with further details about subgroup analyses about the design of electronic health records (EHRs)

## Abbreviations

df: degrees of freedom
EHR: Electronic health record
FET: Fisher’s exact text
Max: Maximum
Mdn: Median
Min: Minimum
SD: Standard deviation
