# Supplementary material for: How should electronic health records be designed? A cross-sectional study in patients with psoriasis
Source: BMC Med Inform Decis Mak. 2019 Nov 12;19:218. doi: 10.1186/s12911-019-0926-5 (PMC6849227; doi:10.1186/s12911-019-0926-5)
Supplement: Supplementary file 1 — Additional File 1. Patient questionnaire (German original language). [file 12911_2019_926_MOESM1_ESM.pdf]

1 Angaben zu Ihrer Person

1.1 Bitte geben Sie Ihr Geschlecht an.

- o männlich
- o weiblich

1.2 Bitte geben Sie Ihr Geburtsjahr an.

Jahr: ___________

1.3 Welchen höchsten schulischen Abschluss haben Sie?

- O noch Schüler (noch kein Abschluss)
- O Fachhochschulreife/ Fachoberschule
- O Schule beendet ohne Abschluss
- O Abitur, allgemeine oder fachgebundene Hochschulreife
- O Haupt-/ Volksschule
- O anderen Schulabschluss (z.B. im Ausland erworben)
- O Mittlerer Schulabschluss/ Realschule/ mittlere Reife/POS

2 Angaben zu Ihrer Psoriasis

2.1 Wann traten das erste Mal Symptome aufgrund der Psoriasis auf?

vor ________ Jahren **oder** im Jahr ___________

2.2 Wann wurde bei Ihnen zum ersten Mal die Diagnose Psoriasis gestellt?

vor ________ Jahren **oder** im Jahr ___________

**2.3**	**Wie häufig gehen Sie aktuell wegen Ihrer Psoriasis zu einem Arzt?**

- ○ jeden Tag
- ○ mind. alle 6 Monate
- ○ mind. 1x pro Woche
- ○ mind. 1x pro Jahr
- ○ mind. 1x pro Monat
- ○ seltener als 1x pro Jahr
- ○ mind. alle 3 Monate

2.4 Wo werden Sie aktuell wegen Ihrer Psoriasis behandelt?
Sie können mehrere Antwortmöglichkeiten ankreuzen.

- ☐ Hausarztpraxis
- ☐ stationäre Behandlung
- ☐ dermatologische Facharztpraxis
- ☐ keine Behandlung
- ☐ dermatologische Facharztklinik

2.5 Wo wurden Sie jemals wegen Ihrer Psoriasis behandelt?
Sie können mehrere Antwortmöglichkeiten ankreuzen.

- ☐ Hausarztpraxis
- ☐ stationäre Behandlung
- ☐ dermatologische Facharztpraxis
- ☐ keine Behandlung
- ☐ dermatologische Facharztklinik

2.6 An welchen weiteren Erkrankungen leiden Sie derzeit laut Diagnose neben der Psoriasis?
Sie können mehrere Antwortmöglichkeiten ankreuzen.

- ☐ Psoriasis Arthritis
- ☐ nicht-alkoholische Fettleberkrankheit
- ☐ Erkrankungen des Herz-Kreislaufsystems (z.B. Bluthochdruck, Arteriosklerose)
- ☐ Chronisch entzündliche Darmerkrankungen (z.B. Morbus Crohn)
- ☐ Diabetes mellitus Typ 2
- ☐ Alkoholabusus
- ☐ Adipositas
- ☐ Nikotinabusus
- ☐ Fettstoffwechselstörungen
- ☐ keine
- ☐ Depression

3 Ihre persönliche Mediennutzung

3.1 Wie häufig nutzen Sie die folgenden internetfähigen Geräte?

	täglich	4-6x pro Woche	1-3x pro Woche	seltener als 1x pro Woche	gar nicht
Stand-Computer	○	○	○	○	○
Laptop	○	○	○	○	○
Tablet-PC	○	○	○	○	○
Smartphone	○	○	○	○	○

3.2 Für welche dieser Aktivitäten nutzen Sie das Internet?
Sie können mehrere Antwortmöglichkeiten ankreuzen.

- ☐ Informationssuche
- ☐ E-Mails schreiben und lesen
- ☐ Unterhaltungsmedien (z.B. Musik, Filme)
- ☐ Social Media (z.B. Facebook, Twitter,)
- ☐ Online-Shopping
- ☐ Apps
- ☐ Online-Banking
- ☐ gar nicht
- ☐ Buchung von Aktivitäten/ Übernachtungen/ Flügen
- ☐ andere, und zwar: _____________

3.3 Aus welchem Anlass nutzen Sie das Internet als Informationsquelle im Bereich Gesundheit?
Sie können mehrere Antwortmöglichkeiten ankreuzen.

- ☐ Daten und Fakten zu Krankheiten
- ☐ Zuwendung und Bestätigung bei Problemen
- ☐ Daten und Fakten zu neuen Therapien
- ☐ Austausch mit anderen Betroffenen
- ☐ Informationen zu Medikamenten
- ☐ gar nicht
- ☐ Rat und Hilfe bei gesundheitlichen Problemen
- ☐ andere, und zwar: _____________

4 Patientenberichtete Informationen

Bei der Behandlung der Psoriasis werden klinische Daten vom Arzt erfasst (z.B. Blutdruck). Es können aber auch patientenberichtete Informationen erfasst werden. Dabei handelt es sich um Angaben über das persönliche Empfinden des Patienten. Diese werden **direkt vom Patienten selbst** in Fragebögen oder Befragungsinstrumente angegeben und können auch von niemandem anderen angegeben werden.

Beispiele für patientenberichtete Informationen sind: Symptome, Schmerz, Juckreiz und Nebenwirkungen, aber auch Lebensqualität, Therapiezufriedenheit, Präferenzen, psychische Belastungen und Gesundheitsverhalten.

4.1 Inwieweit stimmen Sie den folgenden Aussagen zu?

	Ja	Nein
Ich gebe bei Arztbesuchen wegen der Psoriasis bestimmte patientenberichtete Informationen an	O *(weiter mit 4.2)*	O
Ich wäre bereit, bei Arztbesuchen wegen der Psoriasis bestimmte patientenberichtete Informationen anzugeben	O	O

4.2 Inwieweit stimmen Sie den folgenden Aussagen zu?

	Stimme voll und ganz zu	Stimme eher zu	Weder noch	Stimme eher nicht zu	Stimme überhaupt nicht zu
Patientenberichtete Informationen können meinem Arzt helfen, über die Psoriasis hinaus weitere Beschwerden und Erkrankungen zu erkennen	O	O	O	O	O
Angaben über patientenberichtete Informationen können bei Entscheidungen über die Behandlung helfen	O	O	O	O	O
Das Beantworten von patientenberichteten Informationen kann mir helfen, meinen subjektiven Leidensdruck besser zu erkennen und einzuordnen	O	O	O	O	O
Ich befürchte, dass Fragebögen über patientenberichtete Informationen mein persönliches Empfinden nicht richtig widerspiegeln können	O	O	O	O	O
Ich würde mir wünschen, dass Entscheidungen über die Behandlung von meinem Arzt und mir gemeinsam getroffen werden	O	O	O	O	O

5 Spezifische patientenberichtete Informationen

Eine wichtige patientenberichtete Information ist die **Lebensqualität** des Patienten. Diese umfasst unterschiedliche Komponenten, wie z.B. *Symptome und Gefühle des Patienten*; *Auswirkungen der Erkrankung auf Freizeitaktivitäten*, *Sport*, *Arbeit oder zwischenmenschliche Kontakte* oder *Probleme mit der Behandlung*.

5.1 **Inwieweit stimmen Sie den folgenden Aussagen zu?**		
	**Ja**	**Nein**
Ich beantworte für meinen Arzt Fragebögen über meine **Lebensqualität**	O *(weiter mit 5.2)*	O
Ich wäre bereit, für meinen Arzt Fragebögen über meine **Lebensqualität** zu beantworten	O	O

5.2 **Inwieweit stimmen Sie den folgenden Aussagen zu?**					
	**Stimme voll und ganz zu**	**Stimme eher zu**	**Weder noch**	**Stimme eher nicht zu**	**Stimme überhaupt nicht zu**
Angaben über meine **Lebensqualität** können Entscheidungen über die Behandlung unterstützen	O	O	O	O	O
Informationen über meine **Lebensqualität** sind mir zu persönlich, um diese mit meinem Arzt zu teilen	O	O	O	O	O
Die Psoriasis hat große Auswirkungen auf meine **Lebensqualität**	O	O	O	O	O

Weitere patientenberichtete Informationen sind z.B. die **persönlichen Therapieziele** und der **empfundene Therapienutzen** des Patienten. Diese können abzielen auf Bereiche wie *soziale Einschränkungen*; *körperliche Einschränkungen*; *mentale Belastungen*; *Einschränkungen durch die Therapie* und *Zuversicht in die Heilung*.

5.3 **Inwieweit stimmen Sie den folgenden Aussagen zu?**		
	**Ja**	**Nein**
Ich beantworte für meinen Arzt Fragebögen über meine **persönlichen Therapieziele**	O *(weiter mit 5.4)*	O
Ich wäre bereit, für meinen Arzt Fragebögen über meine **persönlichen Therapieziele** zu beantworten	O	O

5.4 **Inwieweit stimmen Sie den folgenden Aussagen zu?**					
	**Stimme voll und ganz zu**	**Stimme eher zu**	**Weder noch**	**Stimme eher nicht zu**	**Stimme überhaupt nicht zu**
Angaben über meine **persönlichen Therapieziele** können Entscheidungen über die Behandlung unterstützen	O	O	O	O	O
Informationen über meine **persönlichen Therapieziele** sind mir zu persönlich, um diese mit meinem Arzt zu teilen	O	O	O	O	O

5.5 **Inwieweit stimmen Sie den folgenden Aussagen zu?**		
	**Ja**	**Nein**
Ich beantworte für meinen Arzt Fragebögen über den von mir **empfundenen Therapienutzen**	O *(weiter mit 5.6)*	O
Ich wäre bereit, für meinen Arzt Fragebögen über den von mir **empfundenen Therapienutzen** zu beantworten	O	O

5.6 **Inwieweit stimmen Sie den folgenden Aussagen zu?**					
	**Stimme voll und ganz zu**	**Stimme eher zu**	**Weder noch**	**Stimme eher nicht zu**	**Stimme überhaupt nicht zu**
Angaben über den von mir **empfundenen Therapienutzen** können Entscheidungen über die Behandlung unterstützen	O	O	O	O	O
Informationen über den von mir **empfundenen Therapienutzen** sind mir zu persönlich, um diese mit meinem Arzt zu teilen	O	O	O	O	O

Wenn Sie noch Anmerkungen zu patientenberichteten Informationen haben, können Sie diese hier eintragen:

6 Eintragung von Informationen in die elektronische Gesundheitsakte

In der elektronischen Gesundheitsakte können unterschiedliche Informationen über einen Patienten gespeichert und jederzeit angeschaut werden. Diese könnten durch den Arzt, den Patienten oder eine andere berechtigte Person in die elektronische Gesundheitsakte eingegeben werden. Der Patient kann entscheiden, wer seine Akte einsehen darf und wofür die gespeicherten Informationen verwendet werden. Mögliche Informationen, die darin gespeichert werden können, sind z.B. *Geschlecht, Geburtsdatum*, *Diagnosen*, *eingesetzte Therapien und Medikamente, Blutwerte*, *Angaben zur Lebensqualität*, *Symptomen* und *Nebenwirkungen.*

6.1 **Inwieweit stimmen Sie den folgenden Aussagen zu?**		
	**Ja**	**Nein**
Ich fülle Papierfragebögen vor/bei Arztbesuchen aus	○	○
Ich fülle elektronische Fragebögen vor/bei Arztbesuchen aus	○	○

6.2	**Sollten die folgenden Personen Zugriff auf Ihre Informationen über die Psoriasis haben?**				
	**Ja, immer**	**Nur, wenn ich es erlaube**	**Nur, wenn ich dabei bin**	**Nein, niemals**	**Mir egal**
Der Arzt, der meine Psoriasis **hauptsächlich** behandelt	O	O	O	O	O
Andere Ärzte, die an der Behandlung meiner Psoriasis **beteiligt** sind (z.B. Hausarzt, Dermatologe und Rheumatologe)	O	O	O	O	O
Andere Leistungserbringer, die an der Behandlung meiner Psoriasis **beteiligt** sind (z.B. Pflegekraft, Ergotherapeut)	O	O	O	O	O
Andere Ärzte, die **nicht** an der Behandlung meiner Psoriasis **beteiligt** sind (z.B. Hausarzt, Orthopäde)	O	O	O	O	O
Andere Leistungserbringer, die **nicht** an der Behandlung meiner Psoriasis **beteiligt** sind (z.B. Physiotherapeut, Logopäde)	O	O	O	O	O
Meine Krankenkasse	O	O	O	O	O

6.3	**Inwieweit stimmen Sie den folgenden Aussagen zu?**					
	**Stimme voll und ganz zu**	**Stimme eher zu**	**Weder noch**	**Stimme eher nicht zu**	**Stimme überhaupt nicht zu**	**Mir egal**
Ich als Patient sollte darüber entscheiden können, wer Zugriff auf meine Daten hat	O	O	O	O	O	O
Ich als Patient sollte wissen, wo und wie meine Daten gespeichert werden	O	O	O	O	O	O
Informationen über mich als Patienten sollten nur von dazu berechtigten Personen in die elektronische Gesundheitsakte eingetragen werden dürfen	O	O	O	O	O	O
Ich wäre damit einverstanden, dass meine Daten anonymisiert der Wissenschaft/ Forschung zur Verfügung stehen	O	O	O	O	O	O

6.4	**Inwieweit treffen die folgenden Aussagen auf Sie zu?**				
	**Trifft voll und ganz zu**	**Trifft eher zu**	**Weder noch**	**Trifft eher weniger zu**	**Trifft überhaupt nicht zu**
Ich kann mir vorstellen, **Informationen** in eine elektronische Gesundheitsakte einzutragen	O	O	O	O	O
Ich kann mir vorstellen, **patientenberichtete Informationen** in eine elektronische Gesundheitsakte einzutragen	O	O	O	O	O

6.5 **Wie häufig wären Sie bereit, Informationen in die elektronische Gesundheitsakte einzutragen?**

○ gar nicht ○ seltener als zu jedem Arztbesuch ○ zu jedem Arztbesuch ○ häufiger als zu jedem Arztbesuch

6.6 **Wie könnten Sie sich vorstellen, Informationen in eine elektronische Gesundheitsakte einzutragen?**

	Trifft voll und ganz zu	Trifft eher zu	Weder noch	Trifft eher weniger zu	Trifft überhaupt nicht zu
von zu Hause aus	○	○	○	○	○
im Wartezimmer	○	○	○	○	○
über mein eigenes Smartphone/Tablet	○	○	○	○	○
über meinen eigenen Laptop/PC	○	○	○	○	○
über ein mir zur Verfügung gestelltes Endgerät (z.B. Smartphone, Tablet, Laptop)	○	○	○	○	○

6.7 **Welche der folgenden Personen sollte klinische Daten (z.B. Blutdruck) in die elektronische Gesundheitsakte eingeben können?**
Sie können mehrere Antwortmöglichkeiten ankreuzen.

☐ Patient ☐ Arzt ☐ nicht-ärztliches Personal

6.8 **Welche der folgenden Personen sollte patientenberichtete Informationen (z.B. Juckreiz, Lebensqualität, Therapieziele) in die elektronische Gesundheitsakte eingeben können?**
Sie können mehrere Antwortmöglichkeiten ankreuzen.

☐ Patient ☐ Arzt ☐ nicht-ärztliches Personal

6.9 **Wie viele Minuten dürfte das Eintragen von Informationen maximal dauern, damit Sie dies…**

	Das wäre mir zu häufig	1 Min.	5 Min.	10 Min.	20 Min.	30 Min.	über 30 Min.
…täglich machen?	○	○	○	○	○	○	○
…wöchentlich machen?	○	○	○	○	○	○	○
…monatlich machen?	○	○	○	○	○	○	○

6.10	**Welche der folgenden Aussagen müssen gegeben sein, damit Sie sich vorstellen können, selber Informationen in die elektronische Gesundheitsakte einzutragen?**				
	**Stimme voll und ganz zu**	**Stimme eher zu**	**Weder noch**	**Stimme eher nicht zu**	**Stimme überhaupt nicht zu**
Die Informationen müssen so übersichtlich gespeichert und dargestellt werden, dass sie bei der Behandlung berücksichtigt werden können	O	O	O	O	O
Der Arzt muss die Informationen aus der elektronischen Gesundheitsakte bei Entscheidungen über die Behandlung berücksichtigen	O	O	O	O	O

Wenn Sie noch Anmerkungen zur Eintragung von Informationen in die elektronischen Gesundheitsakte haben, können Sie diese hier eintragen:

7 Einstellungen zur elektronischen Gesundheitsakte

7.1	**Wie wichtig sind Ihnen die folgenden Aussagen? Durch die elektronische Gesundheitsakte…**				
	**Sehr wichtig**	**Eher wichtig**	**Weder noch**	**Eher unwichtig**	**Sehr unwichtig**
…kann mein Arzt Informationen über mich übersichtlich und schnell abrufen	O	O	O	O	O
…kann mein Arzt auch patientenberichtete Informationen über mich berücksichtigen	O	O	O	O	O
…kann die Kommunikation und Zusammenarbeit zwischen allen an der Behandlung der Psoriasis Beteiligten verbessert werden	O	O	O	O	O
…kann ich als Patient selbst Informationen über mich und meine Krankheit einsehen	O	O	O	O	O

7.2 Inwieweit stimmen Sie den folgenden Aussagen zu? Durch die elektronische Gesundheitsakte…					
	**Stimme voll und ganz zu**	**Stimme eher zu**	**Weder noch**	**Stimme eher nicht zu**	**Stimme überhaupt nicht zu**
…kann die Kommunikation mit meinem Arzt verbessert werden	O	O	O	O	O
…kann die Beziehung zu meinem Arzt verbessert werden	O	O	O	O	O
…kann der Verlauf meiner Erkrankung über einen längeren Zeitraum hinweg beobachtet werden	O	O	O	O	O
…kann ich mich während der Arztbesuche besser an meine Symptome und mein Wohlbefinden erinnern	O	O	O	O	O
…kann ich mehr Kontrolle über meine Erkrankung erhalten	O	O	O	O	O

7.3 Wie realistisch schätzen Sie die folgenden Aussagen ein?					
	**Sehr realistisch**	**Eher realistisch**	**Weder noch**	**Eher un-realistisch**	**Sehr un-realistisch**
Die Nutzung der elektronischen Gesundheitsakte kann den Aufwand für mich als Patienten während des Arztbesuchs verringern	O	O	O	O	O
Die Nutzung der elektronischen Gesundheitsakte kann den Aufwand für mich als Patienten insgesamt verringern	O	O	O	O	O
Die Nutzung der elektronischen Gesundheitsakte kann die Qualität der Behandlung insgesamt verbessern	O	O	O	O	O

Wenn Sie noch Anmerkungen zur elektronischen Gesundheitsakte haben, können Sie diese hier eintragen:

8 Darstellung von Ergebnissen

Informationen, die in der elektronischen Gesundheitsakte gespeichert sind, können in Grafiken oder Tabellen übersichtlich und verständlich dargestellt werden. Die Darstellung der Daten könnte so aussehen:

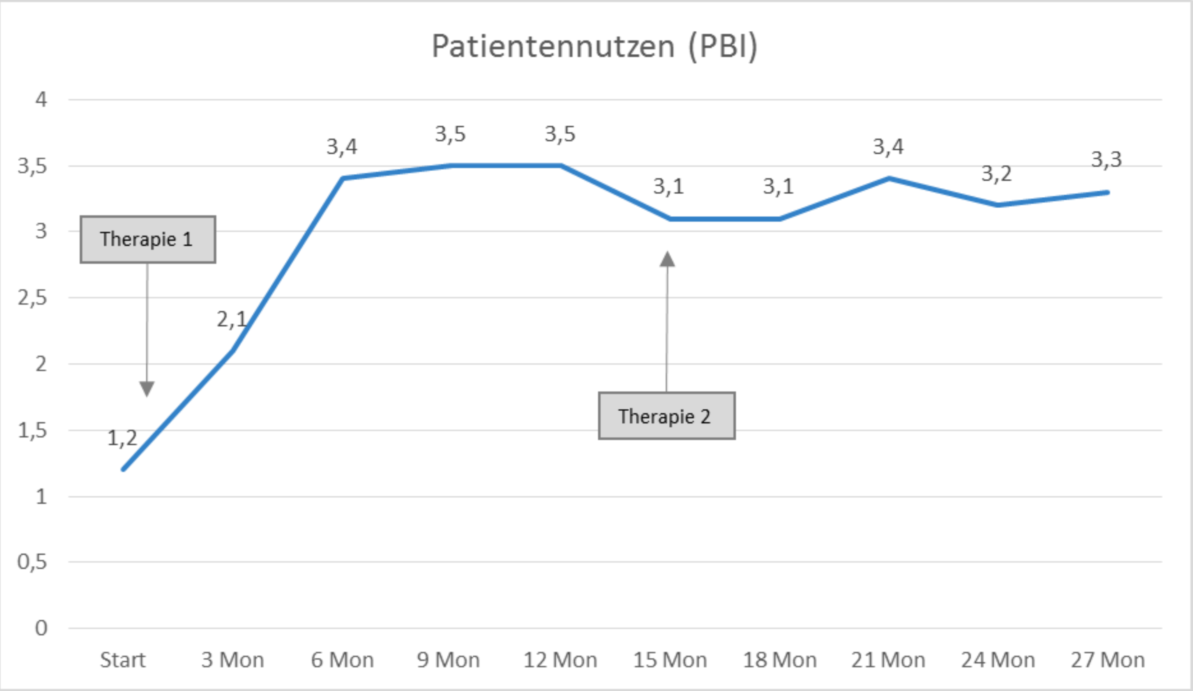

Zeitpunkt der Dateneingabe	Start	3 Mon	6 Mon	9 Mon	12 Mon	15 Mon	18 Mon	21 Mon	24 Mon	27 Mon	Ø	Spanne/Range
Patientennutzen (PBI)	1.2	2.1	3.4	3.5	3.5	3.1	3.1	3.4	3.2	3.3	**3.0**	0-4
Patientennutzen nach Priorität (PBI)	Start	3 Mon	6 Mon	9 Mon	12 Mon	15 Mon	18 Mon	21 Mon	24 Mon	27 Mon		
schmerzfrei zu sein												verbessert
keinen Juckreiz mehr zu empfinden												unverändert
kein Brennen an der Haut mehr zu haben												verschlechtert
von allen Hausveränderungen geheilt zu sein												
besser schlafen zu können												
weniger niedergeschlagen zu sein												
an Lebensfreude zu gewinnen												
keine Frucht vor einem Fortschreiten der Krankheit zu haben												
ein normales Alltagsleben führen zu können												
im Alltag leistungsfähiger zu sein												
Ihre Angehörigen und Freunde weniger zu belasten												
normalen Freizeitaktivitäten nachgehen zu können												
ein normales Berufsleben führen zu können												
mehr Kontakte mit anderen Menschen haben zu können												
sich mehr zeigen zu mögen												
in der Partnerschaft weniger belastet zu sein												
ein normales Sexleben führen zu können												
weniger auf Arzt- und Klinikbesuche angewiesen zu sein												
weniger Zeitaufwand mit der täglichen Behandlung zu haben												
weniger eigene Behandlungskosten zu haben												
weniger Nebenwirkungen zu haben												
eine klare Diagnose und Therapie zu finden												
Vertrauen in die Therapie zu haben												
eine schnellere Verbesserung der Haut zu erfahren												
eine Kontrolle über Ihre Erkrankung zurpck zu gewinnen												
Verbessert (n=)	20	20	18	22	17	21	25	21	18	21	**20.3**	0-25
Unverändert (n=)	5	3	5	2	6	4	4	2	5	3	**3.9**	0-25
Verschlechtert (n=)	0	2	2	1	2	0	1	2	2	1	**1.3**	0-25
Patientennutzen (PBI)	1.2	2.1	3.4	3.5	3.5	3.1	3.1	3.4	3.2	3.3	**3.0**	0-4
Körperoberfläche und Schweregrad (PASI; %)	52	46	72	81	41	71	55	72	81	65	**63.6**	0-100
Lebensqualität (DLQI)	5	3	7	5	3	5	3	7	5	4	**4.7**	0-32
Status Nagelbeteiligung (NAPPA-Clin, %)	89	92		81	64	62	69	81		94	**79.0**	0-100
Nagelspezifische Lebensqualität (NAPPA-QoL; %)	81	85		92	45	78	72	75		92	**77.5**	0-100
Nagelspezifischer Nutzen (NAPPA-PBI; %)	92	89		84	51	71	69	86		91	**79.1**	0-100
Schmerzen (VAS; %)	65	81		42	92				84	88	**75.3**	0-100

8.1 **Wie wichtig sind die folgenden Aussagen Ihrer Ansicht nach?**					
	**Sehr wichtig**	**Eher wichtig**	**Weder noch**	**Eher unwichtig**	**Sehr unwichtig**
Ich kann mir meine Daten in Grafiken oder Tabellen selbst ansehen	O	O	O	O	O
Mein Arzt kann sich meine Daten in Grafiken oder Tabellen ansehen	O	O	O	O	O
Die grafische Darstellung meiner Daten dient als gemeinsame Besprechungsgrundlage für mich und meinen Arzt	O	O	O	O	O
Die grafische Darstellung meiner Daten sollte das Arzt-Patienten-Gespräch unterstützen, darf es aber nicht ersetzen	O	O	O	O	O

8.2 **Inwieweit stimmen Sie den folgenden Aussagen zu?**					
	**Stimme voll und ganz zu**	**Stimme eher zu**	**Weder noch**	**Stimme eher nicht zu**	**Stimme überhaupt nicht zu**
Ich würde meine Daten auch ohne eine grafische Darstellung verstehen und brauche diese daher nicht	O	O	O	O	O
Um zu wissen, wie es mir geht, vertraue ich lieber auf meinen Körper als auf die Darstellung von Daten in Grafiken oder Tabellen	O	O	O	O	O
Es wäre für mich belastend meine Daten anzusehen, da ich dadurch noch mehr über meine Erkrankung nachdenken würde	O	O	O	O	O

Wenn elektronische Gesundheitsakten von unterschiedlichen Patienten vorliegen, können die Daten eines Patienten mit den Ergebnissen von einer Vielzahl anderer Patienten verglichen werden. Dadurch kann eingeschätzt werden, wie der jeweilige Patient im Vergleich zu anderen Patienten in ähnlicher Situation dasteht.

8.3 **Inwieweit stimmen Sie den folgenden Aussagen zu?**					
	**Stimme voll und ganz zu**	**Stimme eher zu**	**Weder noch**	**Stimme eher nicht zu**	**Stimme überhaupt nicht zu**
Ich fände es informativ, meine **klinischen**, krankheitsbezogenen Daten mit den Daten von anderen Patienten zu vergleichen	O	O	O	O	O
Ich fände es informativ, meine Angaben über **patientenberichtete Informationen** mit den Daten von anderen Patienten zu vergleichen	O	O	O	O	O
Es wäre für mich **beruhigend**, meine Daten mit den Daten von anderen Patienten zu vergleichen	O	O	O	O	O
Es wäre für mich **beunruhigend**, meine Daten mit den Daten von anderen Patienten zu vergleichen	O	O	O	O	O
Durch einen Vergleich meiner Daten mit den Daten von anderen Patienten wäre ich informierter und könnte mit meinem Arzt besser über meine Behandlung sprechen	O	O	O	O	O
Sollte eine elektronische Gesundheitsakte eingeführt werden, würde ich nicht wollen, dass meine Daten anonym in Vergleiche mit anderen Patienten einfließen	O	O	O	O	O

Wenn Sie noch weitere Anmerkungen haben, können Sie diese hier eintragen:

Vielen Dank für Ihre Teilnahme!
