# Supplementary material for: How should electronic health records be designed? A cross-sectional study in patients with psoriasis
Source: BMC Med Inform Decis Mak. 2019 Nov 12;19:218. doi: 10.1186/s12911-019-0926-5 (PMC6849227; doi:10.1186/s12911-019-0926-5)
Supplement: Supplementary file 2 — Additional File 2. Tables S1-S5 with further details about subgroup analyses about the design of electronic health records (EHRs) [file 12911_2019_926_MOESM2_ESM.pdf]

Additional File 2: Tables S1-S5 with further details about subgroup analyses about the design of electronic health records.

Table S1: Cross table between age and the design of electronic health records

Table S2: Cross table between level of education and the design of electronic health records

Table S3: Cross table between burden of patient and the design of electronic health records

Table S4: Cross table between number of internet activities and the design of electronic health records

Table S5: Cross table between ever having used electronic questionnaires and the design of electronic health records

Table S6: Cross table between mode of administration and the design of electronic health records

Table S1: Cross table between age and the design of electronic health records

		Age ≤ 40 years	41 – 60 years	≥ 61 years	p-values
I can think of entering data in the waiting room (n=184)					< 0.001, X²
Totally/rather applies	[n (%)]	**35 (74.5)**	32 (41.0)	**16 (27.1)**	< 0.001, FET
	Residuals	**4.7**	-1.0	**-3.4**	
Neither nor	[n (%)]	2 (4.3)	3 (3.8)	5 (8.5)	
	Residuals	-0.4	-0.8	1.2	
Applies rather not/ not at all	[n (%)]	**6 (12.8)**	29 (37.2)	**27 (45.8)**	
	Residuals	**-3.5**	0.9	**2.4**	
Missing value	[n (%)]	4 (8.5)	14 (17.9)	(11) 18.6	
	Residuals	-1.6	0.7	0.7	
I can think of entering data using own smartphone or tablet (n=184)					0.010, X²
Totally/rather applies	[n (%)]	**37 (78.7)**	48 (61.5)	**28 (47.5)**	0.012, FET
	Residuals	**2.8**	0.0	**-2.7**	
Neither nor	[n (%)]	1 (2.1)	1 (1.3)	**6 (10.2)**	
	Residuals	-0.9	-1.7	**2.7**	
Applies rather not/ not at all	[n (%)]	**5 (10.6)**	17 (21.8)	**18 (30.5)**	
	Residuals	**-2.1**	0.0	**2.0**	
Missing value	[n (%)]	4 (8.5)	12 (15.4)	7 (11.9)	
	Residuals	-1.0	1.0	-0.2	
I can think of entering data using a provided device (n=184)					0.008, X²
Totally/rather applies	[n (%)]	**32 (68.1)**	35 (44.9)	**17 (28.8)**	0.006, FET
	Residuals	**3.6**	-0.2	**-3.2**	
Neither nor	[n (%)]	2 (4.3)	3 (3.8)	5 (8.5)	
	Residuals	-0.4	-0.8	1.2	
Applies rather not/ not at all	[n (%)]	**9 (19.1)**	26 (33.3)	**26 (44.1)**	
	Residuals	**-2.4**	0.0	**2.2**	
Missing value	[n (%)]	4 (8.5)	14 (17.9)	11 (18.6)	
	Residuals	-1.6	0.7	0.7	
Maximum duration for entering data on a daily basis (n=184)					0.004, X²
20 minutes and more	[n (%)]	0 (0.0)	2 (2.6)	1 (1.7)	0.001, FET
	Residuals	-1.0	0.9	0.0	
1 to 10 minutes	[n (%)]	**24 (51.1)**	20 (25.6)	15 (25.4)	
	Residuals	**3.2**	-1.6	-1.3	
Daily entry seen as too often	[n (%)]	23 (48.9)	48 (61.5)	31 (52.5)	
	Residuals	-1.0	1.4	-0.5	
Missing value	[n (%)]	**0 (0.0)**	8 (10.3)	**12 (20.3)**	
	Residuals	**-2.8**	-0.2	**2.8**	
Patient should be able to decide who has access to data (n=181)					0.005, X²
Totally/rather applies	[n (%)]	**42 (89.4)**	75 (98.7)	58 (100)	0.006, FET
	Residuals	**-3.3**	1.3	1.7	
Neither nor	[n (%)]	**5 (10.6)**	1 (1.3)	0 (0.0)	
	Residuals	**3.3**	-1.3	-1.7	
Applies rather not/ not at all	[n (%)]	**-**	-	-	
	Residuals	**-**	-	-	

Cells with significant standardized, adjusted residuals marked bold; Percentages sum up to 100% per column; X²: Chi-square test; FET: Fisher's exact test.

Table S2: Cross table between level of education and the design of electronic health records

		Level of education			
		Low	Middle	High	p-values
I can think of entering data at home (n=183)					0.003, X^2
Totally/rather applies	[n (%)]	22 (73.3)	34 (64.2)	77 (77.0)	0.001, FET
	Residuals	0.1	-1.7	1.4	
Neither nor	[n (%)]	2 (6.7)	**4 (7.5)**	**0 (0.0)**	
	Residuals	1.1	**2.1**	**-2.7**	
Applies rather not/ not at all	[n (%)]	1 (3.3)	5 (9.4)	18 (18.0)	
	Residuals	-1.7	-0.9	2.1	
Missing value	[n (%)]	5 (16.7)	**10 (18.9)**	**5 (5.0)**	
	Residuals	1.1	**2.2**	**-2.8**	
I can think of entering data using own smartphone or tablet (n=183)					0.012, X^2
Totally/rather applies	[n (%)]	16 (53.3)	31 (58.5)	66 (66.0)	0.008, FET
	Residuals	-1.0	-0.6	1.3	
Neither nor	[n (%)]	3 (10.0)	3 (5.7)	2 (2.0)	
	Residuals	1.6	0.5	-1.7	
Applies rather not/ not at all	[n (%)]	3 (10.0)	10 (18.9)	26 (26.0)	
	Residuals	-1.7	-0.5	1.7	
Missing value	[n (%)]	**8 (26.7)**	9 (17.0)	**6 (6.0)**	
	Residuals	**2.5**	1.1	**-2.9**	
I can think of entering data using own laptop or computer (n=183)					0.016, X^2
Totally/rather applies	[n (%)]	15 (50.0)	30 (56.6)	**71 (71.0)**	0.011, FET
	Residuals	-1.7	-1.2	**2.3**	
Neither nor	[n (%)]	3 (10.0)	3 (5.7)	2 (2.0)	
	Residuals	1.6	0.5	-1.7	
Applies rather not/ not at all	[n (%)]	3 (10.0)	9 (17.0)	19 (19.0)	
	Residuals	-1.1	0.0	0.8	
Missing value	[n (%)]	**9 (30.0)**	11 (20.8)	**8 (8.0)**	
	Residuals	**2.4**	1.3	**-3.0**	
I can think of entering data using a provided device (n=183)					0.010, X^2
Totally/rather applies	[n (%)]	13 (43.3)	26 (49.1)	44 (44.0)	0.009, FET
	Residuals	-0.2	0.6	-0.4	
Neither nor	[n (%)]	**4 (13.3)**	2 (3.8)	4 (4.0)	
	Residuals	**2.1**	-0.6	-1.0	
Applies rather not/ not at all	[n (%)]	**4 (13.3)**	15 (28.3)	**42 (42.0)**	
	Residuals	**-2.5**	-0.9	**2.7**	
Missing value	[n (%)]	**9 (30.0)**	10 (18.9)	**10 (10.0)**	
	Residuals	**2.3**	0.7	**-2.4**	
Maximum duration for entering data on a daily basis (n=183)					0.031, X^2
20 minutes and more	[n (%)]	**2 (6.7)**	1 (1.9)	0 (0.0)	0.026, FET
	Residuals	**2.4**	0.2	-1.9	
1 to 10 minutes	[n (%)]	12 (40.0)	18 (34.0)	29 (29.0)	
	Residuals	1.0	0.3	-1.0	
Daily entry seen as too often	[n (%)]	12 (40.0)	25 (47.2)	**64 (64.0)**	
	Residuals	-1.8	-1.4	**2.6**	
Missing value	[n (%)]	4 (13.3)	9 (17.0)	7 (7.0)	
	Residuals	0.5	1.7	-1.9	
Maximum duration for entering data on a monthly basis (n=183)					0.009, X^2
	[n (%)]	8 (26.7)	12 (22.6)	30 (30.0)	0.006, FET

20 minutes and more	Residuals	-0.1	-0.9	0.9	
1 to 10 minutes	[n (%)]<br>Residuals	**11 (36.7)**<br>**-2.3**	29 (54.7)<br>-0.2	62 (62.0)<br>1.9	
Monthly entry seen as too often	[n (%)]<br>Residuals	2 (6.7)<br>0.7	3 (5.7)<br>0.5	3 (3.0)<br>-1.0	
Missing value	[n (%)]<br>Residuals	**9 (30.0)**<br>**3.1**	9 (17.0)<br>1.1	**5 (5.0)**<br>**-3.4**	
Access to data: other physician not treating the psoriasis (n=174)					0.028, X²<br>0.018, FET
Yes, always	[n (%)]<br>Residuals	**15 (57.7)**<br>**2.4**	21 (42.9)<br>1.0	**28 (28.3)**<br>**-2.7**	
After permission or patient being present	[n (%)]<br>Residuals	**9 (34.6)**<br>**-2.8**	27 (55.1)<br>-0.7	**67 (67.7)**<br>**2.6**	
No, never	[n (%)]<br>Residuals	2 (7.7)<br>1.0	1 (2.0)<br>-0.8	4 (4.0)<br>0.0	
Access to data: health insurance company (n=176)					0.014, X²<br>0.013, FET
Yes, always	[n (%)]<br>Residuals	15 (53.6)<br>1.5	**27 (55.1)**<br>**2.4**	**30 (30.3)**<br>**-3.2**	
After permission or patient being present	[n (%)]<br>Residuals	11 (39.3)<br>-1.0	20 (40.8)<br>-1.1	53 (53.5)<br>1.7	
No, never	[n (%)]<br>Residuals	2 (7.1)<br>-0.8	2 (4.1)<br>-1.9	**16 (16.2)**<br>**2.3**	

Cells with significant standardized, adjusted residuals marked bold; Percentages sum up to 100% per column; X²: Chi-square test; FET: Fisher's exact test.

Table S3: Cross table between burden of patient and the design of electronic health records

		Burden of patient		
		Low	High	p-values
I can think of entering data at home (n=187)				0.013, X²
Totally/rather applies	[n (%)]	**90 (66.2)**	**45 (88.2)**	0.010, FET
	Residuals	**-3.0**	**3.0**	
Neither nor	[n (%)]	6 (4.4)	0 (0.0)	
	Residuals	1.5	-1.5	
Applies rather not/ not at all	[n (%)]	20 (14.7)	5 (9.8)	
	Residuals	0.9	-0.9	
Missing value	[n (%)]	**20 (14.7)**	**1 (2.0)**	
	Residuals	**2.5**	**-2.5**	
Maximum duration for entering data on a weekly basis (n=187)				0.035, X²
20 minutes and more	[n (%)]	12 (8.8)	6 (11.8)	0.020, FET
	Residuals	-0.6	0.6	
1 to 10 minutes	[n (%)]	72 (52.9)	34 (66.7)	
	Residuals	-1.7	1.7	
Weekly entry seen as too often	[n (%)]	**38 (27.9)**	**4 (7.8)**	
	Residuals	**2.9**	**-2.9**	
Missing value	[n (%)]	14 (10.3)	7 (13.7)	
	Residuals	-0.7	0.7	
Maximum duration for entering data on a monthly basis (n=187)				0.031, X²
20 minutes and more	[n (%)]	34 (25.0)	18 (35.3)	0.031, FET
	Residuals	-1.4	1.4	
1 to 10 minutes	[n (%)]	81 (59.6)	23 (45.1)	
	Residuals	1.8	-1.8	
Monthly entry seen as too often	[n (%)]	8 (5.9)	0 (0.0)	
	Residuals	1.8	-1.8	
Missing value	[n (%)]	13 (9.6)	10 (19.6)	
	Residuals	-1.9	1.9	

Cells with significant standardized, adjusted residuals marked bold; Percentages sum up to 100% per column; X²: Chi-square test; FET: Fisher's exact test.

Table S4: Cross table between number of internet activities and the design of electronic health records

		Number of internet activities			
		0 to 3 activities	4 to 6 activities	7 to 9 activities	p-values
I can think of entering data at home (n=187)					0.010, X^2
Totally/rather applies	[n (%)]	**15 (44.1)**	**74 (79.6)**	46 (76.7)	0.008, FET
	Residuals	**-4.0**	**2.2**	0.9	
Neither nor	[n (%)]	2 (5.9)	2 (2.2)	2 (3.3)	
	Residuals	1.0	-0.8	0.1	
Applies rather not/ not at all	[n (%)]	**9 (26.5)**	9 (9.7)	7 (11.7)	
	Residuals	**2.5**	-1.5	-0.5	
Missing value	[n (%)]	**8 (23.5)**	8 (8.6)	5 (8.3)	
	Residuals	**2.5**	-1.1	-0.9	
I can think of entering data in the waiting room (n=187)					0.008, X^2
Totally/rather applies	[n (%)]	**10 (29.4)**	**35 (37.6)**	**39 (65.0)**	0.006, FET
	Residuals	**-2.0**	**-2.0**	**3.8**	
Neither nor	[n (%)]	3 (8.8)	6 (6.5)	1 (1.7)	
	Residuals	1.0	0.7	-1.5	
Applies rather not/ not at all	[n (%)]	15 (44.1)	33 (35.5)	16 (26.7)	
	Residuals	1.3	0.4	-1.5	
Missing value	[n (%)]	6 (17.6)	19 (20.4)	**4 (6.7)**	
	Residuals	0.4	1.8	**-2.3**	
I can think of entering data using own smartphone or tablet (n=187)					< 0.001, X^2
Totally/rather applies	[n (%)]	**8 (23.5)**	60 (64.5)	**47 (78.3)**	< 0.001, FET
	Residuals	**-5.0**	0.8	**3.3**	
Neither nor	[n (%)]	3 (8.8)	3 (3.2)	2 (3.3)	
	Residuals	1.4	-0.7	-0.4	
Applies rather not/ not at all	[n (%)]	**13 (38.2)**	20 (21.5)	**8 (13.3)**	
	Residuals	**2.5**	-0.1	**-2.0**	
Missing value	[n (%)]	**10 (29.4)**	10 (10.8)	**3 (5.0)**	
	Residuals	**3.4**	-0.6	**-2.1**	
I can think of entering data using own laptop or computer (n=187)					0.001, X^2
Totally/rather applies	[n (%)]	**10 (29.4)**	63 (67.7)	**44 (73.3)**	0.001, FET
	Residuals	**-4.4**	1.5	**2.1**	
Neither nor	[n (%)]	2 (5.9)	5 (5.4)	1 (1.7)	
	Residuals	0.5	0.7	-1.2	
Applies rather not/ not at all	[n (%)]	**10 (29.4)**	14 (15.1)	9 (15.0)	
	Residuals	**2.0**	-0.9	-0.7	
Missing value	[n (%)]	**12 (35.3)**	11 (11.8)	6 (10.0)	
	Residuals	**3.5**	-1.4	-1.4	
Maximum duration for entering data on a daily basis (n=187)					0.011, X^2
20 minutes and more	[n (%)]	1 (2.9)	2 (2.2)	0 (0.0)	0.006, FET
	Residuals	0.7	0.6	-1.2	
1 to 10 minutes	[n (%)]	7 (20.6)	32 (34.4)	22 (36.7)	
	Residuals	-1.7	0.5	0.8	
Too often	[n (%)]	17 (50.0)	49 (52.7)	37 (61.7)	
	Residuals	-0.7	-0.7	1.2	
Missing value	[n (%)]	**9 (26.5)**	10 (10.8)	**1 (1.7)**	
	Residuals	**3.3**	0.0	**-2.7**	

Maximum duration for entering data on a weekly basis (n=187)					0.032, X²
20 minutes and more	[n (%)]	3 (8.8)	11 (11.8)	6.7 (4)	0.047, FET
	Residuals	-0.2	1.0	-0.9	
1 to 10 minutes	[n (%)]	**13 (38.2)**	55 (59.1)	38 (63.3)	
	Residuals	**-2.4**	0.7	1.3	
Too often	[n (%)]	9 (26.5)	18 (19.4)	15 (25.0)	
	Residuals	0.6	-1.0	0.6	
Missing value	[n (%)]	**9 (26.5)**	9 (9.7)	3 (5.0)	
	Residuals	**3.1**	-0.7	-1.9	
Patient should be able to decide who has access to data (n=184)					0.001, X²
Totally/rather applies	[n (%)]	33 (100)	**92 (100)**	**53 (89.8)**	0.002, FET
	Residuals	1.2	**2.5**	**-3.6**	
Neither nor	[n (%)]	0 (0.0)	**0 (0.0)**	**6 (10.2)**	
	Residuals	-1.2	**-2.5**	**3.6**	
Applies rather not/ not at all	[n (%)]	-	-	-	
	Residuals	-	-	-	

Cells with significant standardized, adjusted residuals marked bold; Percentages sum up to 100% per column; X²: Chi-square test; FET: Fisher’s exact test.

Table S5: Cross table between ever having used electronic questionnaires and the design of electronic health records

		Ever used electronic questionnaires			
		Yes	No	Missing value	p-values
I can think of entering data at home (n=187)					0.020, X²
Totally/rather applies	[n (%)]	33 (76.7)	97 (73.5)	**5 (41.7)**	0.039, FET
	Residuals	0.8	0.6	**-2.4**	
Neither nor	[n (%)]	2 (4.7)	4 (3.0)	0 (0.0)	
	Residuals	0.6	-0.2	-0.7	
Applies rather not/ not at all	[n (%)]	3 (7.0)	20 (15.2)	2 (16.7)	
	Residuals	-1.4	1.1	0.3	
Missing value	[n (%)]	5 (11.6)	11 (8.3)	**5 (41.7)**	
	Residuals	0.1	-1.9	**3.5**	
Maximum duration for entering data on a daily basis (n=187)					0.060, X²
20 minutes and more	[n (%)]	0 (0.0)	2 (1.5)	1 (8.3)	0.032, FET
	Residuals	-1.0	-0.2	1.9	
1 to 10 minutes	[n (%)]	14 (32.6)	47 (35.6)	**0 (0.0)**	
	Residuals	0.0	1.3	**-2.5**	
Too often	[n (%)]	23 (53.5)	72 (54.5)	8 (66.7)	
	Residuals	-0.2	-0.2	0.8	
Missing value	[n (%)]	6 (14.0)	11 (8.3)	3 (25.0)	
	Residuals	0.8	-1.6	1.7	
Access to data: other service providers treating the psoriasis (n=182)					0.008, X²
Yes, always	[n (%)]	**22 (51.2)**	**31 (24.4)**	2 (16.7)	0.011, FET
	Residuals	**3.4**	**-2.6**	-1.1	
After permission or patient being present	[n (%)]	**21 (48.8)**	90 (70.9)	10 (83.3)	
	Residuals	**-2.8**	1.9	1.3	
No, never	[n (%)]	0 (0.0)	6 (4.7)	0 (0.0)	
	Residuals	-1.4	1.6	-0.7	
Access to data: other physician not treating the psoriasis (n=178)					0.031, X²
Yes, always	[n (%)]	**24 (55.8)**	**37 (29.8)**	5 (45.5)	0.020, FET
	Residuals	**2.9**	**-3.0**	0.6	
After permission or patient being present	[n (%)]	**18 (41.9)**	**82 (66.1)**	5 (45.5)	
	Residuals	**-2.6**	**2.9**	-0.9	
No, never	[n (%)]	1 (2.3)	5 (4.0)	1 (9.1)	
	Residuals	-0.6	0.1	0.9	
Access to data: other service providers not treating the psoriasis (n=182)					0.006, X²
Yes, always	[n (%)]	**16 (38.1)**	**16 (12.9)**	2 (16.7)	0.007, FET
	Residuals	**3.6**	**3.2**	-0.2	
After permission or patient being present	[n (%)]	24 (57.1)	89 (71.8)	8 (66.7)	
	Residuals	-1.7	1.6	-0.1	
No, never	[n (%)]	2 (4.8)	19 (15.3)	2 (16.7)	
	Residuals	-1.8	1.4	0.4	

Cells with significant standardized, adjusted residuals marked bold; Percentages sum up to 100% per column; X²: Chi-square test; FET: Fisher's exact test.

Table S6: Cross table between mode of administration and the design of electronic health records

		Mode of administration		
		Paper-based	Electronic	p-values
I can think of entering data at home (n=187)				0.014, X^2
Totally/rather applies	[n (%)]	**26 (56.9)**	**106 (77.9)**	0.013, FET
	Residuals	**-2.9**	**2.9**	
Neither nor	[n (%)]	1 (2.0)	5 (3.7)	
	Residuals	-0.6	0.6	
Applies rather not/ not at all	[n (%)]	**12 (23.5)**	**13 (9.6)**	
	Residuals	**2.5**	**-2.5**	
Missing value	[n (%)]	9 (17.6)	12 (8.8)	
	Residuals	1.7	-1.7	
I can think of entering data in the waiting room (n=187)				0.049, X^2
Totally/rather applies	[n (%)]	**31 (60.8)**	**53 (39.0)**	0.044, FET
	Residuals	**2.7**	**-2.7**	
Neither nor	[n (%)]	3 (5.9)	7 (5.1)	
	Residuals	0.2	-0.2	
Applies rather not/ not at all	[n (%)]	**11 (21.6)**	**53 (39.0)**	
	Residuals	**-2.2**	**2.2**	
Missing value	[n (%)]	6 (11.8)	23 (16.9)	
	Residuals	Residuals	0.9	

Cells with significant standardized, adjusted residuals marked bold; Percentages sum up to 100% per column; X^2: Chi-square test; FET: Fisher's exact test.
